# 射频消融治疗肺磨玻璃结节的临床价值

**DOI:** 10.3779/j.issn.1009-3419.2021.101.35

**Published:** 2021-10-20

**Authors:** 晓刚 谭, 宝东 刘

**Affiliations:** 100053 北京，首都医科大学宣武医院胸外科 Department of Thoracic Surgery, Xuan Wu Hospital of Capital Medical University, Beijing 100053, China

**Keywords:** 射频消融, 肺磨玻璃结节, 临床疗效, Radiofrequency ablation, Lung ground-glass nodule, Clinical effective

## Abstract

**背景与目的:**

随着计算机断层扫描（computed tomography, CT）广泛应用于肺癌筛查，越来越多的肺磨玻璃结节（ground-glass nodule, GGN）被发现，尽早干预有利于提高肺癌患者的生存率。射频消融（radiofrequency ablation, RFA）是治疗原发性或转移性肺部恶性肿瘤的一种替代方法。本研究旨在探讨RFA治疗肺GGN的安全性和临床疗效。

**方法:**

选择我院2016年6月-2021年3月收治的24例肺GGN患者，共计28枚结节，接受RFA治疗，其中男性13例，女性11例，平均年龄为（69.4±11.1）岁。接受RFA的GGN大小为（1.30±0.56）cm；消融范围为（2.50±0.63）cm；消融时间为（15.00±8.68）min。

**结果:**

全部结节手术顺利，所有患者无围术期死亡，术中无严重并发症发生。中位随访时间为25个月。1例术后2个月因心梗去世。28个结节均无局部进展，局部控制率为100.0%。*Kaplan-Meier*分析患者1年、2年的总体生存率分别为95.8%、95.8%；肿瘤特异生存率分别为100.0%、100.0%。

**结论:**

RFA是治疗肺GGN安全有效的微创技术。

## Abstract (en)

**Background and objective:**

With the wide application of computed tomography (CT) in the screening of early lung cancer, more and more ground glass nodules (GGNs) have been found. Early intervention is helpful to improve the survival rate of lung cancer patients. Radiofrequency ablation (RFA) is an alternative option to manage primary or metastatic lung malignancies. The purpose of this study is to review the safety and clinical efficacy for lung GGN treated by RFA.

**Methods:**

From June 2016 to March 2021, 24 patients with a total of 28 lung GGNs in our hospital underwent 28 sessions of RFA. There were 13 males and 11 females with an average age of (69.4±11.1) years. The size of GGN receiving RFA was (1.30±0.56) cm; The ablation range was (2.50±0.63) cm and ablation time was (15.00±8.68) min.

**Results:**

The procedure of all RFAs went smoothly, no perioperative deaths occurred and no serious complications during the operation. The median follow-up was 25 months. One case died of myocardial infarction 2 months after operation. All 28 GGNs showed no evidence of local progression and the local control rate was 100.0%. *Kaplan-Meier* analysis showed that the 1-year and 2-year overall survival rates were 95.8% and 95.8%; the tumor specific survival rates were 100.0% and 100.0%, respectively.

**Conclusion:**

RFA is a safe, effective and minimally invasive technique for the treatment of lung GGNs.

在世界范围内肺癌发病率虽位居第二，但死亡率仍高居首位^[1]^。2019年我国防癌报告^[2]^显示2015年我国肺癌的发病率为57.26/10万，新发病例约为78.7万例。其发病率在男性、女性分居一、二位。肺癌死亡率为45.87/10万，死亡共计63.1万例，男性和女性肺癌死亡率均为恶性肿瘤的第一位。随着社会经济水平的不断提髙，人们的健康意识不断增强，低剂量螺旋计算机断层扫描（computed tomography, CT）被用于越来越多的高危人群肺癌筛查，肺结节被发现的概率也随之增加^[3]^。肺结节是指直径≤3 cm的肺部病灶，按照其密度均匀与否和实质成分占比，分为纯磨玻璃结节（pure ground-glass nodule, pGGN）、混合磨玻璃结节（mixed GGN, mGGN）和实性结节^[4]^。前两种生长相对缓慢，但较肺实性结节恶变概率高^[5-7]^。此外，如pGGN在生长过程中出现实性成分或增大，此时该结节会有更高的风险转变为浸润性腺癌^[8, 9]^，及早干预有利于提高肺癌患者的生存率。然而，部分可疑恶性肺结节的患者因身体、年龄及合并其他系统疾病等因素，不能耐受或拒绝接受手术等治疗方法^[10, 11]^，因此，需要运用其他合适的治疗手段。在过去的十余年，微创和精准两大理念彻底改变了肺癌诊治的面貌。在微创治疗领域中，主要用于早期非小细胞肺癌（non-small cell lung cancer, NSCLC）的治愈性消融和中晚期NSCLC的姑息性消融^[13, 14]^。

射频消融（radiofrequency ablation, RFA）能在较短时间内使病灶内局部温度升高到60 ℃-100 ℃，当组织被加热至60 ℃以上时，肿瘤细胞内蛋白质立即发生变性、细胞坏死及肿瘤组织灭活，从而达到治疗的目的。研究^[15, 16]^表明RFA在控制NSCLC及肺转移瘤方面均具有临床应用价值。RFA安全，并发症少；适形，效果可靠；微创，患者恢复快；操作简单，可重复进行，适用于多发性肺肿瘤^[17]^。本研究回顾性分析24例肺部GGN采用RFA治疗的安全性和临床疗效，现报道如下。

## 1 资料与方法

### 1.1 一般资料

回顾性分析2016年6月-2021年3月在首都医科大学宣武医院胸外科接受RFA治疗的肺部GGN患者24例，其中男性13例，女性11例；年龄46岁-89岁，平均年龄（69.4±11.1）岁。4例各有2枚结节，共计治疗28枚结节。右肺16枚（右上肺11枚，右中叶2枚，右下叶3枚），左肺12枚（左上肺8枚，左下叶4枚）。pGGN型8枚，mGGN型20枚。吸烟史7例，无吸烟史17例。纳入标准：①不适合或患者拒绝手术或其他局部治疗如放疗；②经病理学证实为恶性肿瘤；③美国东部肿瘤协作组（Eastern Cooperative Oncology Group, ECOG）评分0分-2分；④术前胸部强化CT无纵隔淋巴结转移；⑤所有GGN实性成分与肿瘤比率（consolidation tumor ratio, CTR）小于50%。伴随病史中，多数患者患有一种或两种基础疾病或伴有手术史（表 1）。术前通过纤维支气管镜虚拟导航穿刺活检或术中经皮肺穿刺获取病理，1枚肺神经内分泌癌，27枚肺腺癌。

**1:** 24例磨玻璃结节患者的临床资料 Clinical information of the 24 GGN patients

Variable	Data
Age (yr)	
Mean±SD	69.4±11.1
≥70	14 (58.3%)
< 70	10 (41.7%)
Gender	
Male	13 (54.2%)
Female	11 (45.8%)
Smoking history	
Yes	7 (29.2%)
No	17 (70.8%)
Comorbidities	
Cerebrovascular disease or CI	4 (16.7%)
Coronary artery disease or PCI	4 (16.7%)
Diabetes mellitus	6 (25.0%)
Hypertension	7 (29.2%)
Previous surgery	
Yes	11 (45.8%)
No	13 (54.2%)
PCI: percutaneous coronary intervention; CI: cerebral infarction; GGN: ground-glass nodule.	

### 1.2 方法

#### 1.2.1 RFA

术前患者和家属签署知情同意书。使用RITA产品（RITA射频针StarBurst XL、StarBurst Xli，RITA射频发生器）。常规消毒铺单，2%利多卡因10 mL局部浸润麻醉，采用Siemens 64排螺旋CT对肿瘤进行1 mm薄层扫描以确定穿刺点和穿刺方向，依据“垂直就近”^[18]^的原则确定进针部位，尽量避开叶裂。保留麻醉注射器于胸壁上再次扫描。根据注射器进针角度和深度再次校正穿刺点及穿刺方向，间断进针并重复局部CT扫描直到病灶位置，射频电极尽可能超过肿瘤边缘外0.5 cm-1 cm组织，以便达到完全消融，部分结节（直径1.5 cm以下）单针消融。射频消融后立即行5 mm厚胸部CT检查，评估消融范围及手术并发症。确定无异常后，患者平卧病床送回病房静卧2 h。对症处理咳嗽、咳血及发热等并发症。在完成RFA后次日早晨进行胸片检查，以检查气胸、血胸和胸腔积液等并发症。

#### 1.2.2 结节大小测量

结节大小测量：肺窗下横截面最大长径；结节实性成分测量：采用肺窗和纵隔窗相结合的方法，以肺窗测量为主（测量最大横截面长径）^[19, 20]^；CTR计算：肺窗下结节的横截面实性成分最大长径与结节最大长径之比^[21, 22]^。

#### 1.2.3 随访

在RFA术后1个月、3个月、6个月、9个月和12个月，尽可能对所有患者进行随访，并进行胸部CT增强扫描，以评估局部疗效。此后，每6个月进行一次随访。与治疗后1个月CT图像进行对比，观察结节是否强化及体积大小有无变化。局部疗效评估以消融后4周-6周时的病灶为基线判断疗效^[23]^。①完全消融（出现下列表现任何一项）：病灶消失、完全形成空洞、病灶纤维化（可为疤痕）、实性结节缩小或无变化或增大（但CT扫描无造影剂异常强化征象）、肺不张（肺不张内的病灶CT扫描无造影剂异常强化征象）；②不完全消融（出现下列表现任何一项）：在形成空洞边缘、在病灶纤维化边缘仍有典型的GGN影像学表现；病灶部分纤维化仍存有部分实性成分，且实性部分CT扫描强化或（和）正电子发射型计算机断层显像（positron emission computed tomography, PET）/CT肿瘤有代谢活性；实性结节，大小无变化或增大，且伴CT扫描造影剂有异常强化征象或（和）PET/CT结节有异常代谢活性。缓慢进展或局部进展者可再次消融，远期疗效通过总体生存（overall survival, OS）率和肿瘤特异生存（cancer-specific survival, CSS）率反映。

### 1.3 统计学分析

统计分析采用SPSS 22.0软件包处理。采用*Kaplan-Meier*分析法估计OS率及CSS率。OS从肺RFA开始计算。对于CSS的评估，终点事件分别是癌症相关死亡和任何死亡或癌症复发。局部控制率：完全消融加不完全消融病例数与完全消融加不完全消融加局部进展病例数之比。

## 2 结果

### 2.1 可行性和安全性

24例患者共进行28次RFA，2例行同侧2次RFA，1例为同期同侧2次RFA手术，1例为双侧2次RFA手术。接受RFA的肺部肿瘤大小为0.7 cm-2.5 cm，平均（1.30±0.56）cm；消融时间为10.0 min-30.0 min，平均（15.00±8.68）min；消融范围为1.0 cm-3.0 cm，平均（2.50±0.63）cm（表 2）。射频电极置入术前计划部位，所有肿瘤按计划方案消融。技术成功率为100.0%（28/28）。没有发生与射频消融相关的死亡。在治疗过程中全部患者均有不同程度的发热感及胀痛，6例术中追加利多卡因局麻均顺利完成治疗；3例发生气胸，1例行胸腔闭式引流，48 h后拔除。13例出现痰中带血，术后口服云南白药治疗，2周后门诊复查症状消失。无出血致血胸的严重并发症。24例患者治疗后均顺利出院。

**2:** 28枚接受RFA的GGN的资料 Summary of 28 GGNs by RFA

Variable	Data
Total RFA time (min)	
Mean±SD	15.00±8.68
≥20	11 (39.3%)
< 20	17 (60.7%)
Tumor size (cm)	
Mean±SD	1.30±0.56
≥2	9 (32.1%)
< 2	19 (67.9%)
Histology	
Adenocarcinoma	27 (96.4%)
Neuroendocrine tumor	1 (3.6%)
Lobar location	
RUL	11 (39.3%)
RML	2 (7.1%)
RLL	3 (10.7%)
LUL	6 (21.4%)
LLL	6 (21.4%)
RFA range (cm)	
Mean±SD	2.50±0.63
≥2	18 (64.3%)
< 2	10 (35.7%)
GGN type	
Pure GGN	8 (28.6%)
GGN with solid component	20 (71.4%)
SD: standard deviation; RUL: right upper lobe; RML: right middle lobe; RLL: right lower lobe; LUL: left upper lobe; LLL: left lower lobe.	

### 2.2 随访

24例生存并随诊截止至2021年7月1日，随访2个月-62个月，中位随访时间为25个月。治疗后随诊复查肺部CT，证实病灶区域从模糊渗出到空洞、伴随周围纤维条索形成，目标结节消失。所有结节近期疗效评价均为完全消融（图 1）。所有结节消融后无复发，局部控制率为100.0%，*Kaplan-Meier*分析患者1年、2年的OS率分别为95.8%、95.8%；CSS率分别为100.0%、100.0%。术后口服抗癌中药治疗，定期复查。死亡1例，术后2个月因心梗去世。

**1:**
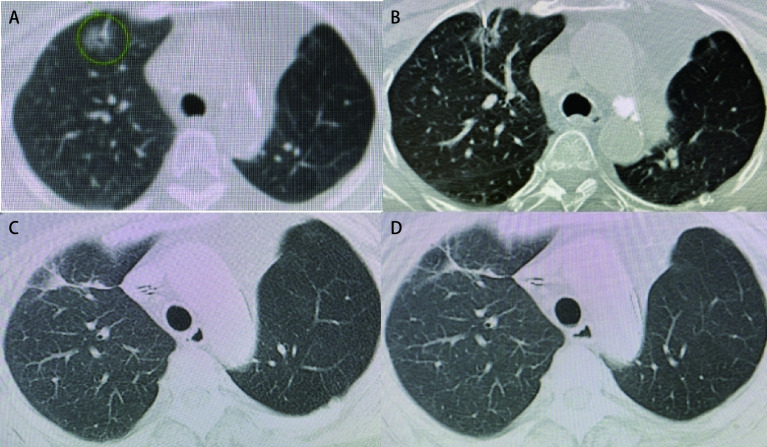
78岁女性，CT发现右肺上叶GGN，组织学病理证实为腺癌。A：射频消融前的CT图像显示右上肺有一个直径2.0 cm的GGN；B：CT透视RFA过程中的图像显示电极被插入GGN；射频消融后10.2个月（C）和26.5个月（D）的CT图像显示消融肿瘤消退，表明完全消融。 Right upper lobe GGN was detected on CT in an 78-year-old woman, histologically confirmed as adenocarcinoma. A: CT image before RFA in the lung window setting shows a tumor, 2.0 cm in diameter, located in the right upper lobe; B: CT images shows that a electrode is introduced into the tumor; 10.2 months (C) and 26.5 months (D) after RFA in the lung window setting shows that the tumor was well controled. CT: computed tomography; RFA: radiofrequency ablation.

## 3 讨论

根据美国国立综合癌症网络（National Comprehensive Cancer Network, NCCN）2020版NSCLC诊治指南，以磨玻璃成分为主的NSCLC首选手术切除，RFA也是可采用的有效手段，尤其针对那些无法耐受或不肯接受手术的患者。RFA已被用于原发性和继发性肺癌的局部治疗，主要用于不适合手术或被认为是手术高危的患者。RFA是治疗无淋巴结转移的GGN型肺癌的一种合适的局部治疗方法。在这项研究中，我们回顾性评估了这些患者RFA的结果。

RFA治疗GGN型肺癌与RFA治疗肺癌实体瘤有一定差异。消融后由于消融区周围的出血、水肿、渗出、炎性细胞的浸润，这种影像学特征将持续3个月-4个月，因此传统的实体瘤疗效评价标准（Response Evaluation Criteria in Solid Tumors, RECIST）不适合用于RFA后局部疗效的评价，特别是GGN^[21]^（图 2）。首先，由于肿瘤周围出现磨玻璃样影（ground-glass opacity, GGO），消融后CT上靶区轮廓变得不清晰。Anderson等^[24]^认为，治疗时缺乏周边毛玻璃光环可能是治疗失败的早期预测因素。对病灶周围的消融区评价不清可能导致治疗失败。RFA后的前3个月，消融病灶的大小通常超过了CT图像上术前肿瘤的大小，因为消融病灶与消融的边缘水肿肺实质混合在一起。在这个时期，RFA的局部控制率不能通过比较肿瘤大小来确定，如有必要，可以做增强CT，通过对比病灶是否增强来确定。也就是说，当整个消融区没有造影增强时，肿瘤被认为是完全治疗的。此后，通过比较先前CT图像中消融区的大小来评估局部疗效。局部进展被定义为消融区的肿瘤进展，当消融区周向扩大或消融区出现规则、散在、结节或偏心病灶时被认为已经发生局部进展。病灶通常表现出一定程度的对比增强，因此与未增强的坏死肿瘤组织形成对比；其次，与实体肺癌不同，PET通常不能用于评估RFA治疗GGN型肺癌的疗效^[25]^；第三，由于肿瘤生长缓慢，在CT上明确发现复发需要更多的时间。pGGN组和mGGN组的肿瘤平均体积倍增时间分别为813 d和457 d^[26]^。一项单中心前瞻性研究^[27]^报道，1.5年局部控制率是93%。只要病灶不侵犯肺门、气管、动脉等重要脏器，经过多次治疗，大多都能完全灭活肿瘤组织。立体定向体部放疗（stereotactic body radiation therapy, SBRT）有时也被用作NSCLC的局部治疗，主要用于不适合手术的患者。Hamamoto等^[28]^报道，28例以GGN型肺癌为主的患者SBRT后2年局部无瘤率为96%。他们的短期结果和我们的相似。而我们28例GGN RFA后随访2个月-62个月，中位随访时间为25个月，并未发现局部复发，可能与GGN肿瘤平均体积倍增时间较长及我们随访时间较短有关，还需要继续随访及时报道相关结果。

**2:**
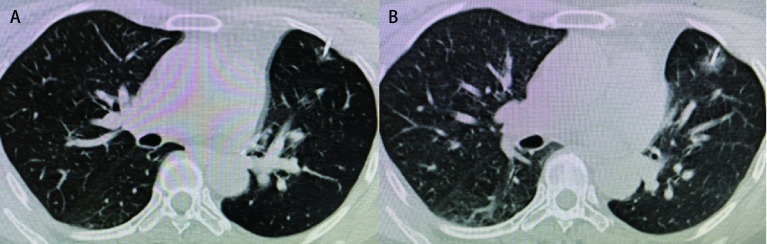
消融前后GGN的CT影像变化。A：消融前GGN；B：消融后肿瘤周围出现GGO，CT上靶区轮廓变得不清晰。 Comparison of CT images of GGNs before and after RFA. A: The image of GGN before RFA was performed; B: The tumor was covered by the GGO after RFA, and the contour of the target area becomes unclear on CT. GGO: ground-glass opacity.

Hiraki等^[29]^报道对临床I期NSCLC行RFA后，患者1年、2年的OS率分别为94%、86%；CSS率分别为100%、93%；无疾病生存率分别为82%、64%，局部疾病进展率为31%。从而得出结论：对于无手术指征的临床I期NSCLC患者，RFA可以最大限度地减轻创伤并使患者生存获益。2015年Iguchi等^[30]^报道了17例RFA治疗GGN合并肺癌的病例，患者1年、5年的OS率分别为93.3%、93.3%；CSS率分别为100%和100%。Kodama等^[31]^报道了42个肺腺癌为主GGN行RFA，共33例患者并非全都为I期，伴随疾病也较多，与我们的研究人群相似，其1年OS率和CSS率均为100%，3年OS率和CSS率分别为96.4%和100%，而我们的结果患者1年、2年的OS率分别为95.8%、95.8%；CSS率分别为100.0%、100.0%，与上述报道的结果接近。Hiraki等^[29]^报道的I期NSCLC患者局部疾病进展率较高，可能是I期NSCLC中GGN占比较低，实性结节RFA复发较早导致。此外，较好的OS率和CSS率可能部分归因于pGGO组和mGGO组的肿瘤生长缓慢，倍增时间超过400 d。这也可显示出RFA在GGN治疗中使患者生存获益。

在系统性回顾研究^[32]^中，与操作有关的并发症发生率为15.2%-55.6%，死亡率为0%-5.6%。最常见的并发症是气胸，发生率为4.5%-61.1%，大部分可以自愈，只有3.3%-38.9%（平均11%）需要放置胸腔闭式引流。本组无操作相关死亡，气胸发生率为12.5%（3/24），其中1例行胸腔闭式引流（4.17%）。本研究小组有丰富的肺穿刺活检经验，避免发生气胸的关键是穿刺技术要熟练，进针速度快和穿刺准确，需要调整射频针的位置时针尖尽量留在肺内或肿瘤内。出现少量气胸不需特殊处理，中、大量气胸需要抽净气体，防止影响射频针在肿瘤内的布针。

RFA与其他疗法相比有一些潜在的优势。RFA可以在局部麻醉下进行，相关的死亡率和发病率较低^[33]^，尽管如此，RFA常用于不适合手术的患者。与传统的外照射治疗或SBRT不同，在检测到局部进展后，重复RFA是可能的^[34]^。重复性是肺RFA的一个显著优点，可以显著改善局部控制结果。在我们的研究中，虽然到随访时间未发现局部进展患者，但是对于肺癌局部进展的患者，重复RFA是可行的。

在高龄或伴随疾病较多的NSCLC的高危患者中，找到手术风险和获益的平衡点非常困难，任何一个方向的微小变化都会显著影响OS，而高危人群在虚弱程度和治疗期望方面是不同的。RFA的耐受性非常好，并且可以重复治疗。综上所述，RFA治疗GGN型肺癌是安全有效的，且有良好的生存率。因此，RFA是肺GGN一种非手术治疗的选择。
